# Evaluation of Benzguinols as Next-Generation Antibiotics for the Treatment of Multidrug-Resistant Bacterial Infections

**DOI:** 10.3390/antibiotics10060727

**Published:** 2021-06-16

**Authors:** Hang Thi Nguyen, Mahmud T. Morshed, Daniel Vuong, Andrew Crombie, Ernest Lacey, Sanjay Garg, Hongfei Pi, Lucy Woolford, Henrietta Venter, Stephen W. Page, Andrew M. Piggott, Darren J. Trott, Abiodun D. Ogunniyi

**Affiliations:** 1 Australian Centre for Antimicrobial Resistance Ecology, School of Animal and Veterinary Sciences, Roseworthy Campus, The University of Adelaide, Roseworthy, SA 5371, Australia; hang.t.nguyen@adelaide.edu.au (H.T.N.); hongfei.pi@adelaide.edu.au (H.P.); 2 Department of Pharmacology, Toxicology, Internal Medicine and Diagnostics, Faculty of Veterinary Medicine, Vietnam National University of Agriculture, Hanoi 100000, Vietnam; 3 Department of Molecular Sciences, Macquarie University, Sydney, NSW 2109, Australia; mahmud.morshed@hdr.mq.edu.au (M.T.M.); elacey@microbialscreening.com (E.L.); andrew.piggott@mq.edu.au (A.M.P.); 4 Microbial Screening Technologies Pty. Ltd., Smithfield, NSW 2164, Australia; dvuong@microbialscreening.com (D.V.); acrombie@microbialscreening.com (A.C.); 5 Clinical and Health Sciences, University of South Australia, Adelaide, SA 5000, Australia; Sanjay.Garg@unisa.edu.au; 6 School of Animal and Veterinary Sciences, Roseworthy Campus, The University of Adelaide, Roseworthy, SA 5371, Australia; lucy.woolford@adelaide.edu.au; 7 Health and Biomedical Innovation, Clinical and Health Sciences, University of South Australia, Adelaide, SA 5000, Australia; rietie.venter@unisa.edu.au; 8 Advanced Veterinary Therapeutics, Newtown, NSW 2042, Australia; swp@advet.com.au

**Keywords:** *Staphylococcus pseudintermedius*, *Staphylococcus aureus*, benzguinols, nidulins, Gram-negative, antimicrobial resistance, colistin, bioluminescent mouse model, cytotoxicity, minimum inhibitory concentration

## Abstract

Our recent focus on the “lost antibiotic” unguinol and related nidulin-family fungal natural products identified two semisynthetic derivatives, benzguinols A and B, with unexpected in vitro activity against *Staphylococcus aureus* isolates either susceptible or resistant to methicillin. Here, we show further activity of the benzguinols against methicillin-resistant isolates of the animal pathogen *Staphylococcus pseudintermedius*, with minimum inhibitory concentration (MIC) ranging 0.5–1 μg/mL. When combined with sub-inhibitory concentrations of colistin, the benzguinols demonstrated synergy against Gram-negative reference strains of *Acinetobacter baumannii*, *Escherichia coli*, *Klebsiella pneumoniae,* and *Pseudomonas aeruginosa* (MICs of 1–2 μg/mL in the presence of colistin), whereas the benzguinols alone had no activity. Administration of three intraperitoneal (IP) doses of 20 mg/kg benzguinol A or B to mice did not result in any obvious adverse clinical or pathological evidence of acute toxicity. Importantly, mice that received three 20 mg/kg IP doses of benzguinol A or B at 4 h intervals exhibited significantly reduced bacterial loads and longer survival times than vehicle-only treated mice in a bioluminescent *S. aureus* murine sepsis challenge model. We conclude that the benzguinols are potential candidates for further development for specific treatment of serious bacterial infections as both stand-alone antibiotics and in combination with existing antibiotic classes.

## 1. Introduction

Infections caused by pathogenic bacteria represent an increasingly significant challenge to public health worldwide [1]. Effective treatment of bacterial infections is becoming increasingly difficult due to the overuse of antibiotics, which has resulted in multidrug-resistance development among many bacterial pathogens [2,3,4]. For example, according to the Centers for Disease Control and Prevention, each year more than 2.8 million multidrug-resistant (MDR) infections occur in the United States, associated with more than 35,000 deaths [5]. It is estimated that, unless urgent action is taken, infections due to pathogens with antimicrobial resistance (AMR) could result in 10 million deaths per year and an economic collapse comparable to the 2008–2009 global financial crisis by 2050 [6,7]. For instance, *Staphylococcus aureus* is the second most clinically important antibiotic-resistant bacterial pathogen in developed countries (behind *Escherichia coli*) and is a major public health concern due to the increasing prevalence of methicillin-resistant *S. aureus* (MRSA) in the hospital environment and within the community [8,9]. The costs associated with MRSA infections are estimated at $10 billion US, averaging about $60,000 per patient [10]. There are also increasing zoonotic risks of methicillin-resistant *Staphylococcus pseudintermedius* (MRSP), i.e., transfer from dogs to owners, making it a potential threat to public health [11,12]. While the situation facing human medicine is dire, the problem is exacerbated in livestock medicine by the limited range of registered drug classes, the risk of transfer of resistance genes through the food chain [13], and the rapid development of pan resistance in one of the most important animal pathogens, enterotoxigenic *E. coli* [14]. The situation is made worse by very few new antibiotics being developed to treat Gram-negative bacterial (GNB) infections [15]. Most newly approved agents against MDR-GNB pathogens are derivatives of existing chemical classes with potential for rapid onset of resistance [16,17]. Polymyxins and some beta lactam–beta lactamase inhibitor combinations including ceftolozane–tazobactam, ceftazidime–avibactam, and meropenem–vaborbactam are used as last resort drug classes for the treatment of GNB infections [18,19].

It is clear that the problems posed by MDR pathogens require rapid development of new, broad-spectrum anti-infectives. Medicinal chemists have been highly successful over the last 50 years in reshaping the scaffolds of earlier antibiotics, both natural and synthetic, including the fourth and fifth generations of β-lactams and third generation of macrolides [20,21,22]. However, significantly new approaches and strategies for breakthrough molecules have not been forthcoming [15,23,24].

**Figure 1:**
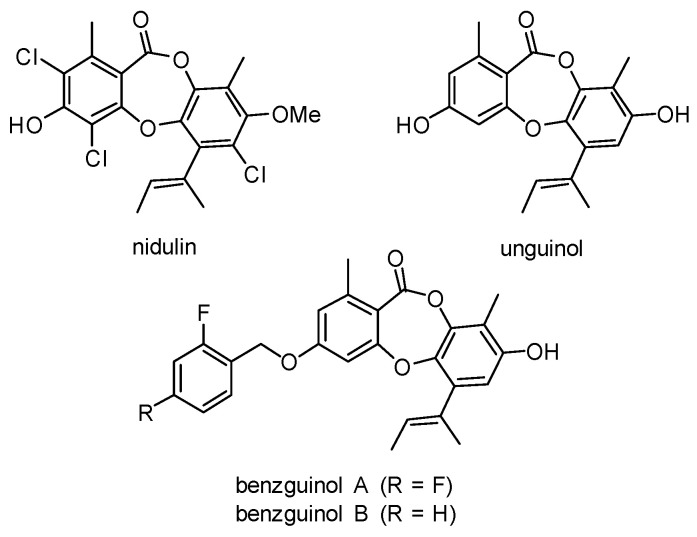
Structures of fungal metabolites nidulin and unguinol and semisynthetic unguinol derivatives benzguinol A and benzguinol B.

As part of our investigations into expanding the chemical space around the “lost antibiotic” nidulin and its related fungal natural products [25], our team recently reported the semisynthesis and in vitro biological evaluation of thirty-four derivatives of the parent fungal depsidone antibiotic, unguinol [26]. Fifteen first-generation unguinol analogs were synthesized and screened against a panel of bacteria, fungi, and mammalian cells to formulate a basic structure–activity relationship for the unguinol pharmacophore. In vitro antibacterial activity testing of these compounds revealed that 3-benzyl analogs, “benzguinols” (Figure 1), 3-*O*-(2,4-difluorobenzyl)unguinol (benzguinol A), and 3-*O*-(2-fluorobenzyl)unguinol (benzguinol B) showed potent activity against both MRSA and methicillin-susceptible *S. aureus* (MSSA) with minimum inhibitory concentration (MIC) ranges of 0.25–1 μg/mL. Based on these results, we concluded that the two compounds are promising candidates for further evaluation of in vivo efficacy [26]. As an extension of that study, we explored the spectrum of activity of benzguinols A and B against the animal health pathogen, MRSP, and examined their potential activity against GNB when combined with sub-inhibitory concentrations of colistin. We also evaluated the efficacy of the benzguinols against *S. aureus* sepsis in a bioluminescent mouse infection model.

## 2. Materials and Methods

### 2.1. Antibiotics and Chemicals Used in this Study

Benzguinol A and benzguinol B were synthesized as described previously [26]. Amikacin, ampicillin, kanamycin, tetracycline, and colistin were purchased from Sigma-Aldrich (Australia). These antibiotics were prepared as 25.6 mg/mL stock solutions as follows: benzguinols A and B were dissolved in 100% DMSO; amikacin, ampicillin, and kanamycin were dissolved in water; and tetracycline was dissolved in 70% ethanol. The antibiotics were aliquoted in 1 mL quantities and stored at −20 °C in the dark.

### 2.2. Organisms and Growth Conditions

Clinical MRSP isolates were obtained from infected skin wounds, ears, abscessed joints, and the urine of dogs as part of the first nation-wide survey on antimicrobial resistance in animal pathogens in Australia [27] at the Australian Centre for Antimicrobial Resistance Ecology (ACARE), School of Animal and Veterinary Sciences, The University of Adelaide, Roseworthy, South Australia. MRSA USA300, *Enterococcus faecalis* ATCC 29212, and 20 porcine vancomycin-resistant enterococci (VRE) were obtained from the University of South Australia collection [28]. Each organism was identified by MALDI-TOF at ACARE before antimicrobial susceptibility testing. For efficacy testing of benzguinol A and benzguinol B, bioluminescent *S. aureus* ATCC 12600 (Xen29; PerkinElmer, Waltham, MA, USA) was used. Reference strains *Acinetobacter baumannii* NCIMB 12457, *A. baumannii* ATCC 19606, *E. coli* ATCC 35218, *E. coli* ATCC 25922; *Klebsiella pneumoniae* ATCC 13883, *K. pneumoniae* ATCC 33495, *Pseudomonas aeruginosa* ATCC 27853, and *P. aeruginosa* PAO1 were provided by SA Pathology, Adelaide, Australia. Bioluminescent *E. coli* Xen14 (a derivative of *E. coli* WS2572) and bioluminescent *P. aeruginosa* Xen41 (a derivative of strain PAO1) were purchased from PerkinElmer Inc (Waltham, MA, USA). The reference strains and clinical isolates were grown on horse blood agar (HBA) and in Luria–Bertani (LB) broth, Miller (Becton Dickinson, Sparks, MD, USA) overnight. *E. coli* Xen14 was grown on HBA containing 30 μg/mL kanamycin, *P. aeruginosa* Xen41 was grown in HBA containing 60 μg/mL tetracycline, and *S. aureus* Xen29 was grown on HBA containing 200 μg/mL kanamycin for selection.

### 2.3. Antimicrobial Susceptibility Testing

The minimum inhibitory concentrations (MICs) of benzguinols A and B against MRSP and selected GNB were determined in round bottom 96-well microtiter plates (Sarstedt 82.1582.001; Mawson Lakes, SA, Australia), using the modified broth micro-dilution method according to recommendations by the Clinical and Laboratory Standards Institute [29] as described previously [30]. Briefly, antimicrobial challenge plates were prepared by serial two-fold dilutions of stock solutions of benzguinol A or B in DMSO. Each dilution was then further diluted 1:100 in LB broth in 96-well plates (Thermo Fisher Scientific, Thebarton, SA, Australia). Benzguinols A and B and amikacin concentrations ranged from 0.03 to 256 μg/mL, and each MIC test was carried out in duplicate and performed on two separate occasions. Negative growth control was LB broth only; positive growth control was bacterial suspension in LB broth. The minimum bactericidal concentration (MBC) was recorded as the lowest concentration of each test compound at which a 99.9% colony count reduction was observed on the plate [31].

Interaction activity between benzguinol A or B with colistin was determined by a modification of the standard checkerboard assay described previously [32,33,34]. Briefly, the antibiotic challenge plates were prepared as described in antimicrobial susceptibility testing. One microliter of benzguinol A or B solution from each combination was dispensed along the abscissa (from Rows A–F) of the 96-well microliter plates and the second compound (colistin) was dispensed along the ordinate (from Columns 3–12) using a 12.5 μL electronic multichannel pipette (VIAFLO Voyager II, Biotools, Loganholme QLD, Australia) followed by addition of 88 μL of LB broth. Thereafter, 10 μL of bacterial suspension were added to each well. One plate was used for each isolate and the plates were incubated at 37 °C for 20 h and observed visually and by *A*_600nm_ measurements. The interaction of two antibiotics was calculated as the fractional inhibitory concentration index (FICI) as described previously [32,33,34] using the following formula:

$$\mathrm{FICI}=\frac{{\mathrm{MIC}}_{A\;\mathrm{in}\;\mathrm{combination}}}{{\mathrm{MIC}}_{A\;\mathrm{alone}}}+\frac{{\mathrm{MIC}}_{B\;\mathrm{in}\;\mathrm{combination}}}{{\mathrm{MIC}}_{B\;\mathrm{alone}}}$$

A is benzguinol A or B, while B is colistin. According to FICI, the interaction between two antibiotic agents was interpreted as follows: synergistic (FICI ≤ 0.5); additive or partially synergistic (0.5 < FICI ≤ 1); indifferent (1 < FICI ≤ 4); and antagonistic (FICI > 4).

The dose reduction index (DRI) was used to describe the difference between the effective dose of benzguinol A or B in combination with colistin from the individual dose of each compound. DRI was calculated using the following formula:

$$\mathrm{DRI}=\frac{{\mathrm{MIC}}_{A\;\mathrm{alone}}}{{\mathrm{MIC}}_{A\;\mathrm{in}\;\mathrm{combination}}}$$

A DRI (>1) is considered beneficial [34,35].

### 2.4. Time-Dependent Growth Inhibitory Assay

The time- and concentration-dependent activities of the benzguinols against one MRSP clinical isolate, one MRSA clinical isolate (and against one reference *E. coli* and one reference *P. aeruginosa* strain in the presence of sub-inhibitory concentrations of colistin) were determined in a kinetics assay by optical density (*A*_600nm_) measurements for 18 h on a Cytation 5 Multimode reader (BioTek, Millennium Science Pty Ltd, Mulgrave, VIC, Australia). Plates for the Gram-positive time-dependent growth inhibitory assays were prepared as described for MIC determinations above, while the plates for the Gram-negative time-dependent growth inhibitory assays were prepared as described for standard checkerboard assays above.

### 2.5. Cytotoxicity Assays

We previously reported the cytotoxicity profiles of benzguinols A and B to Hep G2 (human hepatocellular carcinoma cell line) and HEK293 (human embryonic kidney cell line) [26]. Here, we examined benzguinol A or B in combination with colistin at 0.5 μg/mL for in vitro cytotoxicity using Hep G2 and HEK293 cell lines following the procedure described earlier [28]. Briefly, cell lines were maintained in Dulbecco’s Modified Eagle’s Medium (DMEM; Gibco Cat No: 12430) supplemented with 10% (*vol/vol*) fetal bovine serum (FBS) and 1% (*vol/vol)* PenStrep (100 U/mL penicillin and 100 μg/mL streptomycin) at 37 °C, 5% CO_2_, and passaged every 3 days. Assays were performed in duplicates in black flat bottom 96-well tissue culture trays (Eppendorf Cat No: 0030741013) seeded with ~1.5 × 10^4^ cells per well. After 24 h incubation, the media was removed, the cells were washed once with medium without antibiotics, and fresh medium supplemented with 10% (*vol/vol*) FBS was added. Then, either benzguinol A or B alone or in combination with colistin was added to each well in doubling dilutions starting at the same concentrations used for MIC testing, using wells containing 1% DMSO only and 64 μg/mL ampicillin as controls. The effect of benzguinol alone or in combination with colistin on the viability of each cell line was monitored at 1 h intervals for 20 h at 37 °C in 5% CO_2_ on a Cytation 5 Cell Imaging Multi-Mode Reader (BioTek, Winooski, VT, USA) using the RealTime-Glo^TM^ MT Cell Viability Assay reagent (Promega, Madison, WI, USA).

### 2.6. Agar Well Diffusion Method

Each benzguinol formulation was prepared as a 6 mg/mL solution and daptomycin as a 1.8 mg/mL solution in 20% (*vol/vol*) DMSO in PEG400 (vehicle). All formulations were tested for antibacterial activity using the agar well diffusion method [36] to ensure that the drugs were released from vehicle as a reference for interpretation of in vivo activity in mice. For this assay, several colonies of an overnight HBA culture of *S. aureus* Xen29 were suspended in saline equivalent to 0.5 McFarland standard (*A*_600nm_ = 0.1). A sterile swab was then dipped in the 0.5 McFarland standard bacterial suspension and used to streak over the entire surface of a sterile plate count agar plate. Punch holes were then made on the agar plates using an 8 mm diameter biopsy punch (Livingstone International Pty Ltd., Sydney, NSW, Australia) and a 100 μL equivalent amount of each formulation to a single treatment dose in mice was placed in the well. Agar plates were then incubated at 37 °C for 20 h, and the antimicrobial activity of each drug was determined by measuring and comparing the zone of inhibition with that of vehicle only.

### 2.7. Ethics Statements

To test the safety of the benzguinols and assess their efficacy against challenge with bioluminescent *S. aureus* Xen29, outbred 5–6-week-old male CD1 (Swiss) mice (weighing 25–30 g), obtained from the Laboratory Animal Services breeding facility of the University of Adelaide, were used. Mice had access to food and water throughout the experiment period. The Animal Ethics Committee of The University of Adelaide (approval number S-2015-151) reviewed and approved all animal experiments. The study was conducted in compliance with the Australian Code of Practice for the Care and Use of Animals for Scientific Purposes (8th Edition 2013) and the South Australian Animal Welfare Act 1985.

### 2.8. Safety Testing of Benzguinols A and B Following Parenteral Administration

To ensure a three-dose regimen would be safe to administer to mice, a safety study was conducted using three intraperitoneal (IP) injection of 20 mg/kg benzguinol A or benzguinol B at 4 h intervals to three mice. Three IP doses of vehicle were used as a control. Mice were observed for clinical signs and the data recorded on a Clinical Record Sheet approved by the Animal Ethics Committee of The University of Adelaide. The mice were monitored every 2 h for the first 12 h, and then at 24, 36, 48, and at 72 h post-treatment. At the conclusion of the experiment (72 h post-treatment), mice were humanely killed and sections of liver, kidneys, spleen, lung, and heart were harvested for histopathological examination.

### 2.9. Histopathological Examination

Mouse organs (liver, kidneys, spleen, lungs, and heart) collected from the IP safety experiment were fixed in 10% neutral-buffered formalin (ChemSupply Australia Pty Ltd., Gillman, SA, Australia) and processed routinely, embedded in paraffin blocks, and sectioned to a thickness of 4 μm. Hematoxylin and eosin-stained sections were observed and recorded under light microscopy. Photomicrographs were captured using a DP25 camera and LabSens software (Olympus, Tokyo, Japan).

### 2.10. Efficacy Testing of Benzguinols A and B after IP Challenge of Mice with Bioluminescent Gram-Positive Bacteria (GPB)

For in vivo efficacy testing of benzguinols A and B in a murine bioluminescent infection model, we used mouse-passaged bioluminescent *S. aureus* ATCC strain 12600 (Xen29, PerkinElmer). Bacteria were grown in LB broth at 37 °C to an *A*_600nm_ of 0.5 (1.5 × 10^8^ CFU/mL). Four groups of mice (*n* = 6 mice per group) were infected IP with 3 × 10^7^ CFU of *S. aureus* Xen29 in 200 μL PBS containing 3% hog gastric mucin type III (Sigma Aldrich, St. Louis, MO, USA). The mice were then imaged immediately in both ventral and dorsal positions on the IVIS Lumina XRMS Series III system. At 2 h post-infection, all mice were imaged again as above. Immediately thereafter, mice in Group 1 were injected IP with the drug vehicle only; mice in Groups 2 and 3 were injected with either benzguinol A or benzguinol B at 20 mg/kg IP; and mice in Group 4 were treated with daptomycin at 6 mg/kg IP. Mice were closely monitored for their clinical conditions and then imaged at 4 h post-infection. At 6 and 10 h post-infection, all surviving mice in each group were imaged and immediately followed by an identical drug and vehicle treatment regimen as described above. In addition, 20 μL of blood were withdrawn from the submandibular vein of each mouse at 2, 6, and 8 h post-infection and serial dilutions of the blood samples plated on HBA to estimate bacterial burden. Mice were further monitored frequently for signs of distress and those that had become moribund or showed any evidence of distress were humanely killed by cervical dislocation. At 18, 24, 28, 36, 48, and 72 h post-infection, surviving mice were monitored and further subjected to bioluminescence imaging. In all experiments, signals were collected from a defined region of interest and total flux intensities (photons/s) analyzed using Living Image Software 4.7.2. Differences in median survival times (time to moribund) for mice between groups were analyzed by the log-rank (Mantel–Cox) tests. Differences in luminescence signals and blood counts between groups were compared by Mann–Whitney *U*-tests.

## 3. Results

### 3.1. In Vitro Activity of Benzguinols A and B Alone against GPB and in Combination with Sub-Inhibitory Concentrations of Colistin against GNB

We previously showed potent activity of benzguinols A and B against MRSA and MSSA, with MICs ranging 0.25–1 μg/mL [26]. In this study, benzguinols A and B showed antimicrobial activities against MRSP (MICs of 0.5–1 μg/mL and MBCs of 4–8 μg/mL) in comparison to the control drug amikacin (MICs and MBCs of 8–16 μg/mL) (Table 1). Both benzguinols have activity against *E. faecalis* ATCC 29212 at 8 μg/mL, but MIC ≥16 μg/mL against all the 20 VRE.

The activities of benzguinol A or B alone, colistin alone, benzguinol A + colistin combination, and benzguinol B + colistin combination were tested against 10 reference GNB strains (two *A. baumannii*, three *E. coli*, two *K. pneumoniae*, and three *P. aeruginosa*), and the results are shown in Table 2. Benzguinol A or B alone had no activity against any of the tested GNB; therefore, their MICs were set as 256 μg/mL to calculate FICI and DRI. For colistin alone, its MIC was 1 μg/mL against the *A. baumannii* strains, 0.25 μg/mL against the *E. coli* strains, and 0.5 μg/mL against the *K. pneumoniae* and *P. aeruginosa* strains. However, in combination, the MIC of benzguinol A and benzguinol B was 1–2 μg/mL against all the GNB (a 128–256-fold reduction), whereas the MIC of colistin in the combination was 0.25 μg/mL against the *A. baumannii* strains, 0.06 μg/mL against the *E. coli* strains, and 0.125 μg/mL against the *K. pneumoniae* and *P. aeruginosa* strains, representing a four-fold dose reduction for colistin against all the GNB (Table 2). The FICI of all combinations was 0.25, showing the synergy of the benzguinol–colistin combinations.

### 3.2. Benzguinols A and B Exhibit Time- and Concentration-Dependent Inhibition of Bacterial Growth

**Figure 2:**
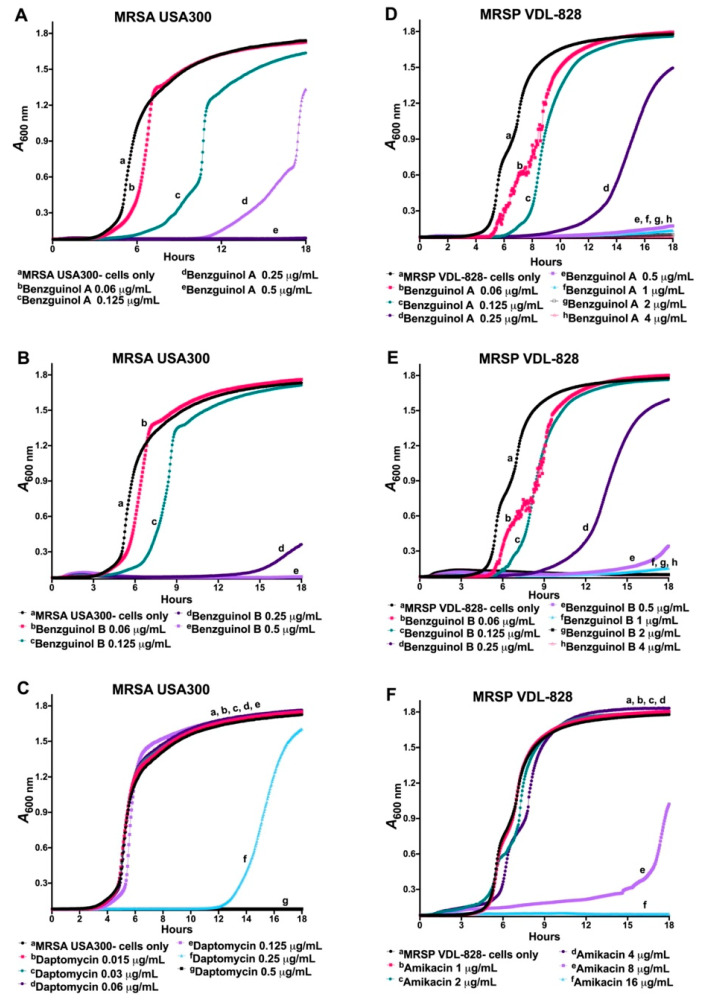
Kinetic assay showing time and concentration-dependent inhibition of MRSA USA300 (**A**–**C**) and MRSP VDL-828 (**D**–**F**) for benzguinol A (**A**,**D**) and benzguinol B (**B**,**E**) using daptomycin (**C**) and amikacin (**F**) as control drugs**.** The sub-minimum inhibitory concentrations for benzguinols A and B = 0.25 μg/mL; daptomycin = 0.25 μg/mL; and amikacin = 8 μg/mL.

The antimicrobial activities of benzguinols A and B were investigated in a time–kill kinetics assay to measure the time and concentration dependent activity of the two compounds against clinical MRSA isolate USA300 and clinical MRSP isolate VDL-828, using daptomycin and amikacin as comparators, respectively. The results show a time- and concentration-dependent inhibition of growth for benzguinols A and B, consistent with features of bacteriostatic drugs. As expected, daptomycin and amikacin displayed patterns of bactericidal drugs (Figure 2).

**Figure 3:**
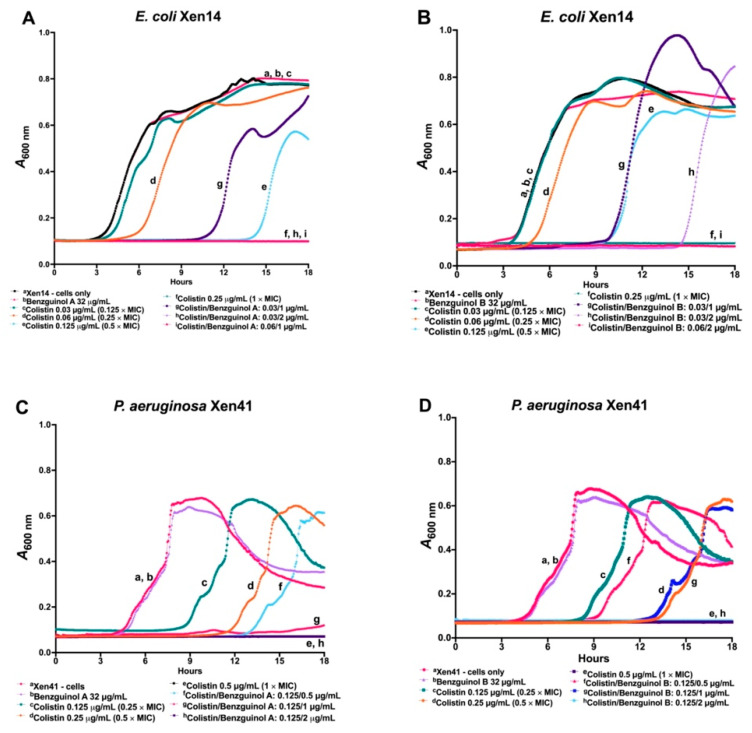
Time- and concentration-dependent antibacterial activities of benzguinols A and B alone and in combination with colistin. Growth inhibitory kinetics of the benzguinols alone or in combination with colistin against *E. coli* Xen14 (**A**,**B**) and *P. aeruginosa* Xen41 (**C**,**D**) were performed on a Cytation 5 Multimode reader (BioTek, Millennium Science Pty Ltd, Mulgrave, VIC, Australia) by optical density (*A*_600nm_) measurements.

The time- and concentration-dependent activities of benzguinols A and B in combination with colistin against GNB was also investigated in a kinetic assay. In this assay, the growth pattern of *E. coli* Xen14 cells treated with benzguinol A (Figure 3A) or benzguinol B (Figure 3B) alone at 32 μg/mL was similar to that of untreated cells. Cells treated with colistin alone at 0.03 μg/mL (0.125 × MIC), 0.06 μg/mL (0.25 × MIC), or 0.125 μg/mL (0.5 × MIC) started to grow at 6, 10, and 14 h, respectively, whereas cells treated with colistin alone at 0.25 μg/mL did not grow. Additionally, cells treated with the benzguinol A + colistin or benzguinol B + colistin combination inhibited bacterial growth more quickly than colistin alone at the same concentration. For example, cells treated with a combination benzguinol A at 1 μg/mL + colistin at 0.03 μg/mL began to grow at around 12 h (approximately 6 h later than colistin alone at 0.03 μg/mL). However, *E. coli* Xen14 cells treated with a benzguinol A at 2 μg/mL + colistin at 0.03 μg/mL combination (Figure 3A) or a combination of benzguinol B at 2 μg/mL + colistin at 0.06 μg/mL (Figure 3B) did not grow.

In a similar time- and concentration-dependent kinetic assay of the combination benzguinol A + colistin or benzguinol B + colistin against *P. aeruginosa* Xen41, the combination with colistin worked more quickly than colistin alone at the same concentration (Figure 3C,D). Xen41 treated with colistin alone at 0.125 μg/mL (0.25 × MIC) and 0.25 μg/mL (0.5 × MIC) started to grow at 9 and 12 h, respectively, whereas cells treated with 0.5 μg/mL colistin alone did not grow. Cells treated with a combination of benzguinol A or benzguinol B at 1 μg/mL + colistin at 0.125 μg/mL started to grow at approximately 13–15 h, which is 4–6 h later than colistin alone, while Xen41 cells treated with a combination of benzguinol A or benzguinol B at 2 μg/mL + colistin at 0.125 μg/mL did not grow. As expected, the growth patterns of Xen41 cells treated with benzguinol A or benzguinol B at 2 μg/mL were similar to those for untreated cells.

### 3.3. Benzguinol A and Benzguinol B in Combination with Colistin Show Low Cytotoxicity to Mammalian Cell Lines

**Figure 4:**
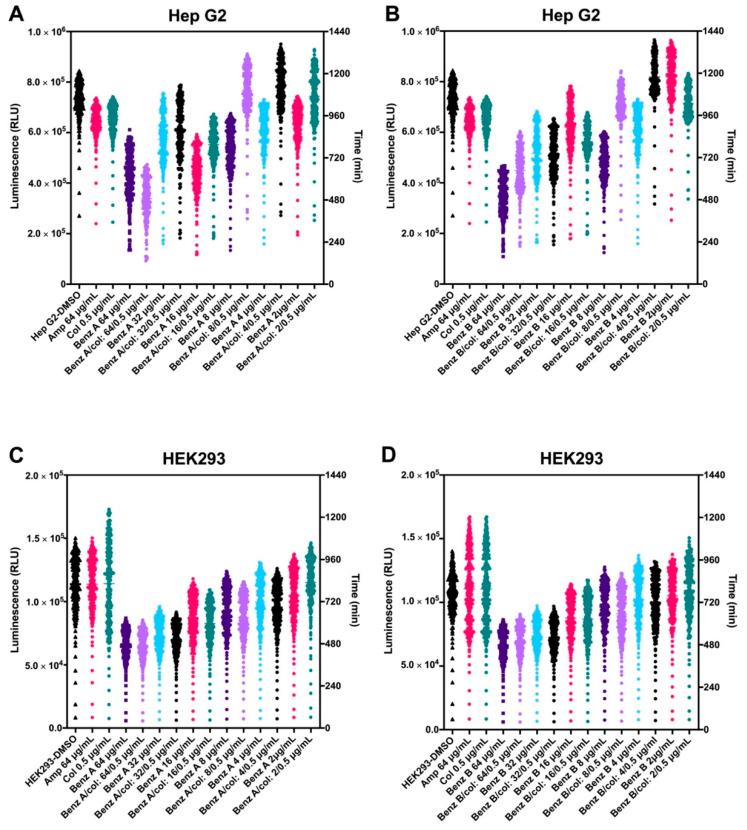
Cytotoxicity assessment of benzguinols A and B alone and in combination with colistin. Real-time cell viability measurements for Hep G2 (**A**,**B**) and HEK293 (**C**,**D**) cells after treatment with different concentrations of benzguinols A and B alone and with 0.5 μg/mL of colistin. The viability of each cell line was measured hourly for 20 h at 37 °C in the presence of 5% CO_2_ on a Cytation 5 Cell Imaging Multi-Mode Reader (BioTek, Millennium Science Pty Ltd, Mulgrave, VIC, Australia) using the RealTime-Glo^TM^ MT Cell Viability Assay reagent (Promega, Madison, WI, USA). Data presented are relative light units (RLU) for each treatment per time point. Abbreviations: Amp, ampicillin; Col, colistin; Benz, benzguinol.

In a previous experiment, we showed that benzguinols A and B demonstrate very low cytotoxicity to Hep G2 (liver) and HEK293 (kidney) cell lines, with both compounds giving IC_50_ value at 32 μg/mL [26]. In this study, we further examined toxicity profiles of the combination of colistin with benzguinol A or benzguinol B to the Hep G2 and HEK293 cell lines (Figure 4). At the concentrations tested, the addition of colistin in the combination did not change the IC_50_ of benzguinol A or benzguinol B. For Hep G2 cells, the presence of colistin appears to reduce the toxicity of benzguinols A and B further (Figure 4A,B).

### 3.4. Benzguinols Show Systemic Safety in Mice

**Figure 5:**
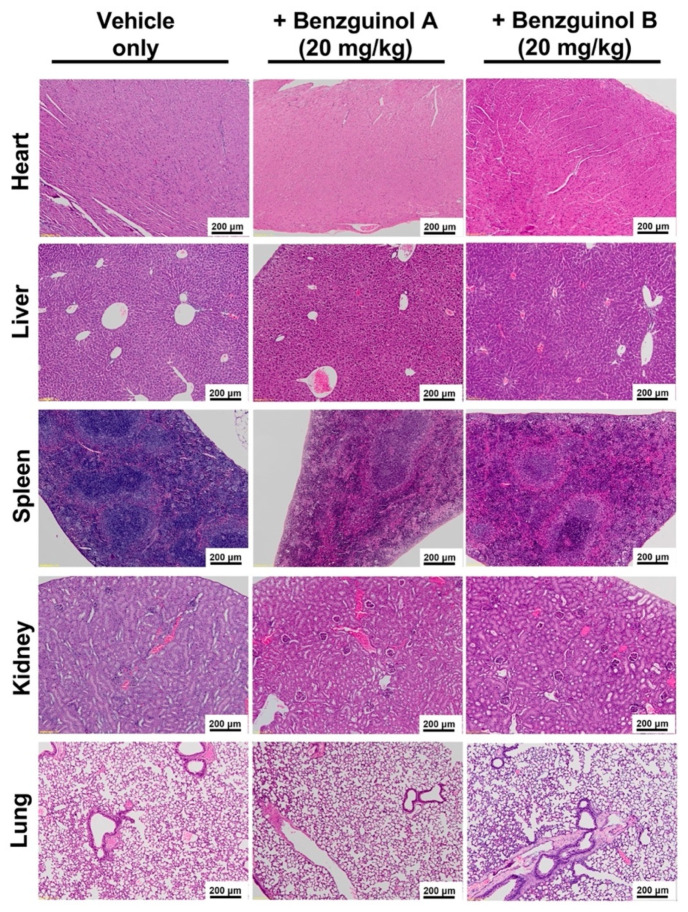
Representative histological images of heart, liver, spleen, lung, and kidneys from benzguinol-treated and control mice harvested at 72 h post-treatment. No morphological abnormalities or changes were observed in mice treated IP with 20 mg/kg benzguinol A, 20 mg/kg benzguinol B, or with vehicle alone. Scale bars: 200 μm.

There were no observable histopathological changes in the liver, heart, spleen, kidneys, and lungs in any mice treated with three IP doses of benzguinol A or benzguinol B at 20 mg/kg (Figure 5).

### 3.5. Agar Well Diffusion Test of Benzguinol Formulations Shows Antibacterial Activity

**Figure 6:**
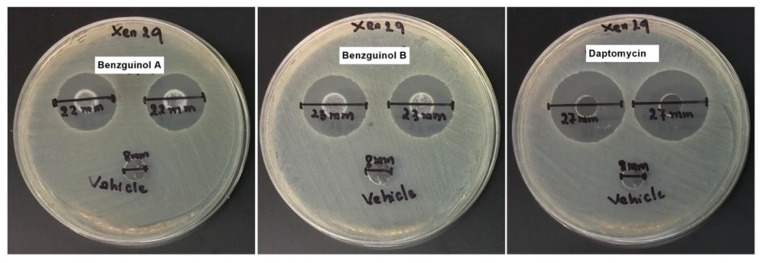
Selected well diffusion of benzguinol A and benzguinol B formulations used in efficacy trial. Each well contained 100 μL of each formulation of benzguinol A or B (600 μg)**,** daptomycin (180 μg) and 100 μL vehicle only. Xen29, bioluminescent *S. aureus* Xen29.

In order to ascertain that the benzguinols are active in the vehicle used, an agar diffusion test of the formulations was carried out. All formulations of benzguinols A and B showed clear inhibitory zones of 22–23 mm, while daptomycin as a control showed an inhibitory zone of 27 mm (Table 3 and Figure 6), indicating that all drugs were released from the vehicle into the agar.

### 3.6. Treatment of Mice with Benzguinol A or Benzguinol B Reduces S. aureus Populations and Significantly Prolongs Survival Times

**Figure 7:**
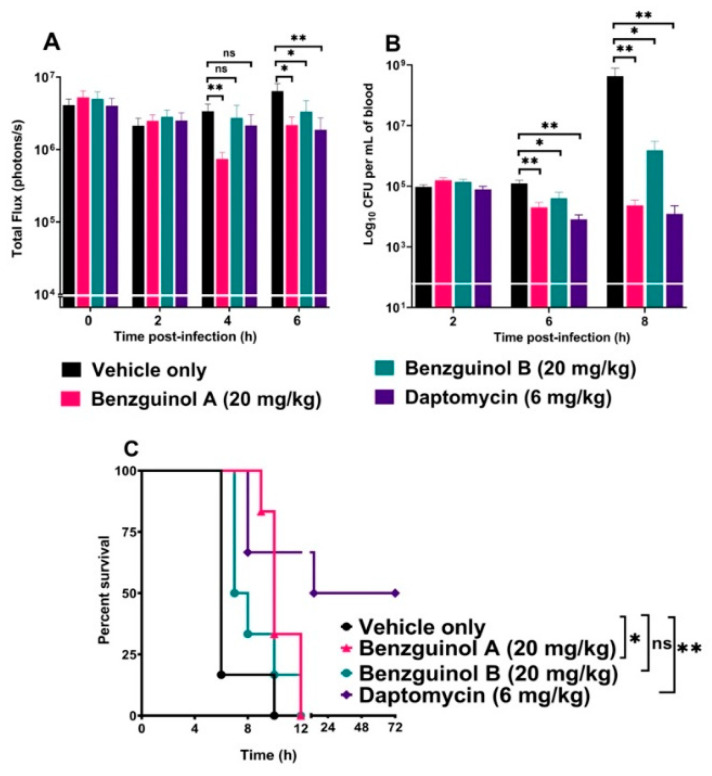
Benzguinol efficacy data. (**A**) Comparison of luminescence signals and (**B**) bacterial load in blood between groups of CD1 mice (*n* = 6) challenged IP with bioluminescent *S. aureus* ATCC 12600 (Xen29) and treated with the indicated drugs. Mice were subjected to bioluminescence imaging on IVIS Lumina XRMS Series III system at the indicated times. ns, not significant; *, *p* < 0.05; **, *p* < 0.01; Mann–Whitney *U*-test (one-tailed). (**C**) Survival analysis for mice treated with the indicated drugs. ns, not significant; *, *p* < 0.05; **, *p* < 0.01; Log-rank (Mantel–Cox test).

The potential of benzguinols A and B as therapeutic drugs against systemic *S. aureus* infection was examined in an IP sepsis challenge model using a well characterized bioluminescent *S. aureus* strain (Xen29). We found that, after the first dose of benzguinol A at 20 mg/kg, there was a statistically significant reduction in *S. aureus* photons at 4 h (*p* = 0.0086, Mann–Whitney *U*-test, one-tailed) and 6 h (*p* = 0.0121, Mann–Whitney *U*-test, one-tailed) (Figure 7A) and significant decrease in number of bacteria at 6 h (*p* = 0.0043, Mann–Whitney test, one-tailed). The second dose of benzguinol A at 6 h post-infection also resulted in significant reduction in bacterial counts at 8 h post-infection (*p* = 0.0022, Mann–Whitney *U*-test, one-tailed) (Figure 7B). Three doses of benzguinol A resulted in significant increase in median survival time compared to the vehicle only control (*p* = 0.017; Mantel–Cox test; Figure 7C).

For benzguinol B, the first dose at 20 mg/kg given at 2 h post-infection resulted in a statistically significant reduction in *S. aureus* photons (*p* = 0.0342, Mann–Whitney *U*-test, one-tailed, Figure 7A), and a significant decrease in the number of bacteria (*p* = 0.0303, Mann–Whitney test, one-tailed) at 6 h post-infection. The second dose of benzguinol B (given at 6 h post-infection) resulted in a significant reduction in the number of bacteria at 8 h post-infection (*p* = 0.0206, Mann–Whitney *U*-test, one-tailed) (Figure 7B) but no significant difference in median survival time compared to the vehicle only control (Figure 7C).

**Figure 8:**
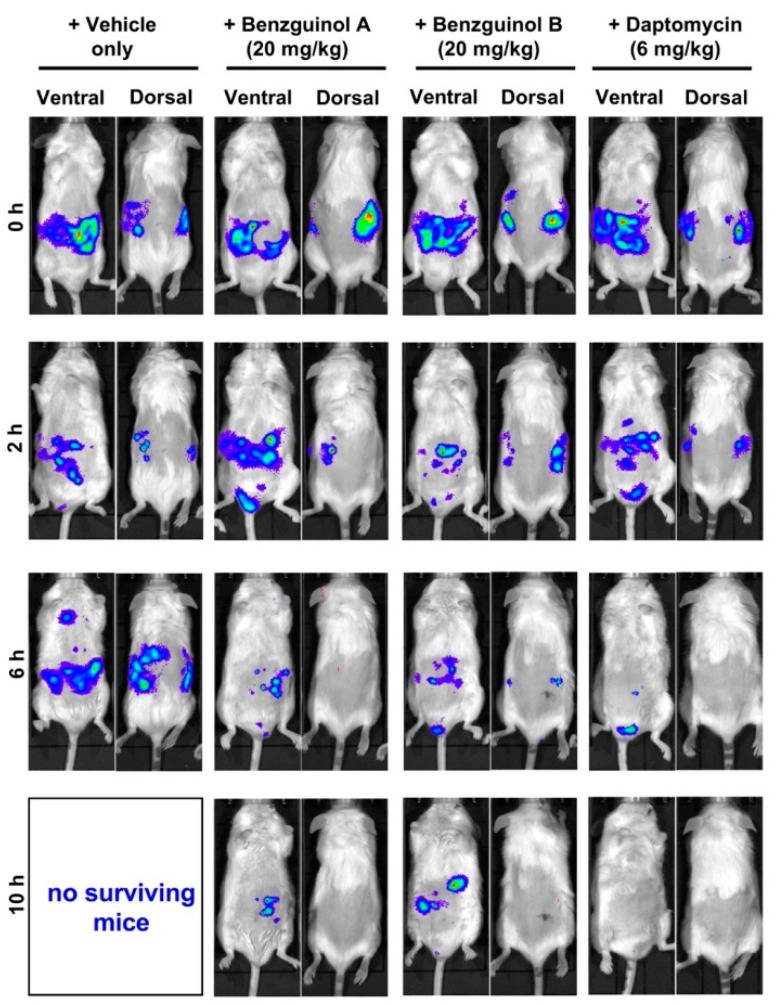
Ventral and dorsal images of representative CD1 mice challenged with approximately 6 × 10^7^ CFU of bioluminescent *S. aureus* ATCC 12600 (Xen29). Mice were treated with benzguinol A or benzguinol B (20 mg/kg), daptomycin (6 mg/kg), or vehicle at 2, 6, and 10 h. Mice were subjected to bioluminescence imaging on IVIS Lumina XRMS Series III system at the indicated times. At 8 h post-infection, all mice treated with vehicle only had become moribund, indicated by “no surviving mice” at 10 h.

Data of photon analysis and bacteria counts are shown up to 6 and 8 h, respectively, due to the number of surviving mice remaining in each group (Figure 7C). The bacterial reduction caused by benzguinols A and B could be clearly observed on images of mice (Figure 8).

## 4. Discussion

The rise in bacterial infections that are resistant to almost all known antibiotics is alarming [37], while at the same time the antibiotic development pipeline has remained stagnant [38]. This global wake-up call has stimulated a debate about how best to combat antibiotic resistance [37]. With this in mind, we have been exploring a strategy involving revisiting some of the old antibiotic scaffolds that were discovered many decades ago but abandoned in favor of more promising leads using modern drug discovery methods to bring new antibiotic classes to the market [39]. In this work, we extended our previous in vitro studies on two semisynthetic analogs of unguinol (benzguinols A and B) [26] to investigate their potential as novel antibiotics for future treatment of bacterial infections.

This study shows three major findings. Firstly, benzguinols A and B demonstrated low MICs against an opportunistic GPB pathogen (MRSP) and also against key strains of GNB (*A. baumannii*, *E. coli*, *K. pneumoniae,* and *P. aeruginosa*) in the presence of sub-inhibitory concentrations of colistin. Secondly, the benzguinols alone or in combination with colistin showed in vitro safety to mammalian (Hep G2 (liver) and HEK293 (kidney)) cell lines and also demonstrated clinical safety in mice with no observed morphological effects on the major organs after three IP doses at 20 mg/kg. Thirdly, treatment of mice with three IP doses of benzguinol A or benzguinol B at 20 mg/kg reduced bioluminescent *S. aureus* populations in vivo and significantly prolonged survival times.

We previously demonstrated that benzguinols A and B show potent activity against MSSA and MRSA at MIC range of 0.25–1 μg/mL (comparable to daptomycin standard). However, unlike daptomycin, the two drugs were shown to have bacteriostatic activity [26]. In this study, we extended our investigation to test the activity of the benzguinols against MRSP clinical isolates and GNB reference strains. The two drugs produced a MIC range of 0.5–1 μg/mL against the MRSP clinical isolates, compared to the amikacin standard with a MIC range of 8–16 μg/mL. Furthermore, while benzguinols A and B alone have no antimicrobial activity against GNB, combination with sub-inhibitory concentrations of colistin resulted in a synergistic interaction when tested against *A. baumannii*, *E. coli*, *K. pneumoniae,* and *P. aeruginosa* ATCC strains, returning MICs of 1–2 μg/mL.

Effective treatment of GNB infections presents a greater challenge than for GPB treatment mainly due to the presence of the outer membrane in GNB, which presents a barrier preventing antibiotic access [40]. Colistin has been shown to interact with the lipopolysaccharide on the surface of GNB. It can then traverse the outer membrane through the self-promoted uptake pathway, resulting in GNB outer membrane disruption [41,42]. For the benzguinol A or benzguinol B + colistin combination, it is hypothesized that the sub-inhibitory concentration of colistin transiently ruptures the outer membrane, thereby allowing passage of the drugs into the cell to reach the drug target site(s). Therefore, a combination of colistin with benzguinol A or benzguinol B could serve as a potential combination for future treatment of bacterial infections. To date, the mode of action of benzguinols is not known or their target(s) identified. However, structure–activity relationship data from our recent work [26] suggest benzguinols and other related family of depsidones may act by binding to a target shared by prokaryotes. Given the lack of information on their target(s), it is quite difficult to speculate the nature of resistance development against benzguinols. As such, identifying the target(s) of benzguinols will be the subject of future investigation.

We previously demonstrated that the benzguinols did not cause hemolysis of human red blood cells (RBCs) at the highest concentration (128 μg/mL) used and both returned IC_50_ values of 32 μg/mL against the HEK293 and Hep G2 cell lines [26]. Further investigation of cell cytotoxicity profiles of combination of benzguinol A or benzguinol B with colistin at 0.5 μg/mL demonstrated no difference in IC_50_ values of either benzguinol alone or in combination with colistin on HEK293 cells and slightly better IC_50_ values against Hep G2 cells. Subsequent in vivo safety testing using three doses of benzguinol A or benzguinol B at 20 mg/kg did not reveal any adverse clinical signs or observable histopathological changes within the main internal mouse organs examined.

Based on the findings above, we investigated the potential of the benzguinols for the treatment of acute sepsis resulting from intraperitoneal inoculation with a bioluminescent derivative of *S. aureus* ATCC 12600 (Xen29). Our results reveal that three IP doses of benzguinol A or B at 20 mg/kg elicited a statistically significant reduction in *S. aureus* populations and prolonged survival times of mice compared to the vehicle-only treated mice. We observed that benzguinol A showed slightly better efficacy, especially in terms of survival percentage. While both compounds have similar MICs of 0.5 μg/mL against *S. aureus* as daptomycin, they were not as effective as daptomycin, associated with a 32-fold increase in MICs in the presence of 10% FBS [26]. This suggests the bioavailability of benzguinols A and B may be quite low in the blood of mice. In addition, the low aqueous solubility of the benzguinols could be a limiting factor in the bioavailability of the drug in the mouse model.

## 5. Conclusions

The study reported here is an extension of previous in vitro investigations of the antibacterial activities of unguinol derivatives benzguinols A and B against GPB pathogens to include evaluation of their potency against MRSP at low concentrations. Our results also show that the combination of benzguinol A or benzguinol B with sub-inhibitory concentrations of colistin resulted in potent activity against key GNB in vitro, suggesting either benzguinol could be combined with colistin for the treatment of GNB infections. In addition, IP treatment of mice with benzguinol A or benzguinol B after systemic *S. aureus* challenge resulted in significant reduction in *S. aureus* populations and prolonged survival times compared to the vehicle-only control, but without clearing the bacterial infection from the bloodstream, suggesting bacteriostatic activity in vivo at the dose administered. Preclinical efficacy testing of a combination of benzguinol A or benzguinol B with colistin is also warranted, potentially overcoming resistance to colistin monotherapy while mitigating toxicity concerned with its use.

Overall, our findings demonstrate that the benzguinols could provide promising new actives evolved from the “lost antibiotic” nidulin family of fungal metabolites for further pharmaceutical and medicinal chemistry development and dose optimization. An intensified effort to enhance the properties of our leads towards improved solubility, reduced plasma binding, and a broader spectrum of action against resistant pathogens is under investigation.

## Figures and Tables

**Table 1:** In vitro activities of benzguinols A and B against methicillin-resistant *Staphylococcus pseudintermedius*.

Compounds	^1^ MIC Range (μg/mL)	^2^ MBC Range (μg/mL)
Benzguinol A	0.5–1	4–8
Benzguinol B	0.5–1	4–8
Amikacin	8–16	8–16

^1^ MIC, minimum inhibitory concentration; ^2^ MBC, minimum bactericidal concentration.

**Table 2:** In vitro activities of benzguinols A and B against Gram-negative bacteria in the presence of sub-inhibitory concentrations of colistin.

Bacteria	MIC (μg/mL)					FICI ^a^		DRI ^b^	
	MIC Alone		MIC in Combination						
	Colistin	Benzguinol A or Benzguinol B	Colistin	Benzguinol A	Benzguinol B	Colistin + Benzguinol A	Colistin + Benzguinol B	Colistin: Benzguinol A	Colistin: Benzguinol B
*A. baumannii* ATCC 19606	1	>256	0.25	2	1	0.25 *	0.25 *	4:128	4:256
*A. baumannii* NCIMB 12457	1	>256	0.25	2	2	0.25 *	0.25 *	4:128	4:128
*E. coli* Xen14	0.25	>256	0.06	1	2	0.25 *	0.25 *	4:256	4:128
*E. coli* ATCC 35218	0.25	>256	0.06	1	2	0.25 *	0.25 *	4:256	4:128
*E. coli* ATCC 25922	0.25	>256	0.06	1	2	0.25 *	0.25 *	4:256	4:128
*K. pneumoniae* ATCC 13883	0.5	>256	0.125	2	2	0.25 *	0.25 *	4:128	4:128
*K. pneumoniae* ATCC 33495	0.5	>256	0.125	2	2	0.25 *	0.25 *	4:128	4:128
*P. aeruginosa* Xen41	0.5	>256	0.125	2	2	0.25 *	0.25 *	4:128	4:128
*P. aeruginosa* PAO1	0.5	>256	0.125	2	2	0.25 *	0.25 *	4:128	4:128
*P. aeruginosa* ATCC 27853	0.5	>256	0.125	2	2	0.25 *	0.25 *	4:128	4:128

MIC, minimum inhibitory concentration. ^a^ FICI, fractional inhibitory concentration index: * synergistic, FICI ≤ 0.5; additive or partially synergistic, 0.5 < FICI ≤ 1; indifferent, 1 < FICI ≤ 4; and antagonistic, FICI > 4. ^b^ DRI, dose reduction index. Bioluminescent *S. aureus* Xen29 was used as the control strain each time the MIC and checkerboard assays were performed; MIC of benzguinol A or B against *S. aureus* Xen29 in each of these assays was 0.5 μg/mL.

**Table 3:** Inhibitory zones of benzguinols A and benzguinol B formulations used for safety and efficacy trials.

Drug	Inhibitory Zone (mm)				
	Safety Trial		Efficacy Trial		
	Vehicle	20 mg/kg	Vehicle	20 mg/kg	6 mg/kg
Benzguinol A	8	23	8	22	ND ^a^
Benzguinol B	8	23	8	23	ND
Daptomycin	8	ND	8	ND	27

^a^ ND, not determined. Each well contained 100 μL of each formulation of benzguinol A or B (600 μg), daptomycin (180 μg), and 100 μL vehicle only.

## Data Availability

The data presented in this study are available on request from the corresponding author. The data are not publicly available due to privacy and access restrictions.
